# Neurocognitive impairment and evidence-based treatment options in Bipolar disorder

**DOI:** 10.1186/s12991-020-00304-4

**Published:** 2020-09-23

**Authors:** Konstantinos N. Fountoulakis

**Affiliations:** 3rd Department of Psychiatry, School of Medicine, Aristotle University of Thessaloniki, 6, Odysseos str (1st Parodos Ampelonon str.), Pylaia, 55535 Thessaloniki, Greece

**Keywords:** Bipolar disorder, Neurocognitive disorder, Cognitive disorder, Cognitive remediation, Functional remediation, treatment

## Abstract

**Background:**

The current paper briefly summarizes the literature on the neurocognitive deficit and its treatment in BD patients.

**Methods:**

The material was chosen on the basis of previous systematic reviews the author has taken part in.

**Results:**

The data so far suggest that the deficit is qualitatively similar but quantitatively milder in comparison to schizophrenia, it is present already since the first episode, is weakly related to mood symptoms and somewhat stronger to psychotic symptoms, it probably determines much of the disability and treatment is problematic. This deficit is also present during periods of euthymia. The possible adverse effect of psychotropic medication is rather small if any at all and is confounded by the specific clinical symptoms, for which medication is used for their treatment. This is especially true concerning antipsychotics and psychotic symptoms. The origin and the etiopathogenesis of the core neurocognitive impairment remain elusive. The presence of a neurodegenerative and of a neurodevelopmental component has both data in favor and against and they are both the focus of debate.

**Conclusions:**

Treatment of the neurocognitive deficit and restoration of functioning is problematic. The data are limited and treatment options are few and with a weak overall effect. Pharmacological treatments, ECT and rTMS present some hard data, while the literature is inconclusive concerning psychotherapeutic interventions.

## Background

The nature of the neurocognitive dysfunction in mood disorders, and particularly in Bipolar disorder (BD), has been the focus of debate for long and only during the last couple of decades the picture became clearer. It seems that the neurocognitive deficit is not only an enduring component of BD, but also represents a core primary characteristic, rather than being secondary to the mood state or medication. A number of other questions are also important, that is whether it is the result of a neurodevelopmental or neurodegenerative process and what is its relationship to the widely believed higher creativity of BD patients.

Today, there is a number of studies suggesting that almost half of BD patients are impaired in one neurocognitive domain, one-third or more are impaired in at least two neurocognitive domains and more than one-fifth in three or more domains [1, 2]. This deficit is rather stable and relatively independent from mood changes, probably reflecting trait features [3–6] Importantly, even after controlling for confounding variables, like education and social class and clinical symptoms, the neurocognitive impairment in BD is less pronounced in comparison to that in schizophrenia [7, 8].

A significant limitation in the literature is that the performance in most tests is influenced by more than one neurocognitive process. This is not a technical problem; it rather reflects the fact that the boundaries between neurocognitive processes are unclear and no process is completely independent from the others. As a consequence, different approaches in their classification and nomenclature have been proposed, adding to the confusion. In Table 1, a list of neurocognitive functions together with the tools used in their assessment are presented.

**Table 1:** Neurocognitive domains assessed in the literature and neuropsychological tools used

Domain	Tool
Premorbid IQ	Single-word reading score from the North American Adult Reading Test (NAARTWide Range Achievement Test (WRAT) Vocabulary subtest score from the Wechsler Adult Intelligence Scale (WAIS)
Current IQ	Wechsler Adult Intelligence Scale (WAIS)
Psychomotor and mental speed	Digit Symbol Substitution Test (DSST) Trail Making Test-A (TMT-A) Reaction time tests
Attention	Continuous Performance Test (CPT) Digits Forward
Working memory	Digits Backward
Verbal memory	
Learning	California Verbal Learning Test (CVLT)
Short delayed recall	Rey Auditory Verbal Learning Test (RAVLT)
Long delayed recall	Wechsler Memory Scale-Logical Memory (WMS-LM)
Recognition	Free recall
Nonverbal memory	Rey Complex Figure Test (RCFT)—Immediate and delayed recall Wechsler Memory Scale-Visual Reproduction (WMS-VR)
Visuospatial function	Block design Rey Complex Figure Test (RCFT)–copy
Language/verbal fluency	Controlled Oral Word Association Test (COWA-FAS) Animal Naming (AN)
Executive function	Wisconsin Card Sorting Test (WCST)—Categories achieved and perseverative errors Stroop Color Word Test (SCWT) Trail Making Test-B (TMT-B)
Social cognition and theory of mind	Benton Facial Recognition Test (BFRT) Faces Test (FT) Eyes Test (ET) Hinting Task (HT) False belief and deception tasks Picture sequencing Character intention tasks Faux Pas

The current paper is a narrative review and reflects the opinion of the author on the issue of the neurocognitive deficit in BD and its treatment options. It is not a systematic review by itself but it is based on the systematic examination of data by the author as this has been published in the past [9, 10]. Essentially it is a selective review updated to date. In this frame, the references included in this paper are also selected as being the most important and thus, the list of references is not exhausted. As the aim was to produce a small comprehensive text, many definitions were omitted and much fundamental knowledge on BD and neurocognition are considered already mastered by the reader.

## General neurocognitive functioning and intelligence quotient (IQ)

The literature suggests that both BD patients and their families have above average IQ and general intellectual functioning [11], or at least they have intelligence similar to healthy controls [12–16].

However, it also suggests that there is a moderate global reduction in comparison to their premorbid state in their neurocognitive functioning as reflected in their IQ scores and their performance in neuropsychological batteries, irrespective of illness phase [17–21].

According to a meta-analysis, patients with BD, show higher Verbal IQ in comparison to Performance IQ scores [22, 23]. The VIQ-PIQ discrepancy might reflect a specific effect of BD on ‘fluid intelligence’ (the capacity to think logically and solve problems in novel situations, independently of acquired knowledge) with a simultaneous respect of the ‘crystallized intelligence’ (the ability to use skills, knowledge, and experience; it does rely on long-term memory) [24].

When all phases of the illness are taken into consideration, the effect sizes concerning current IQ reduction, range from 0.36 to 0.70. For patients in remission, the results of meta-analyses are inconclusive. The effect sizes reported for PIQ range from 0.16 to 0.50 [24, 25].

## Psychomotor and mental speed

Mental speed and psychomotor activation are two concepts which overlap and include reaction time, cognitive and motor speed and, manual dexterity, however, they are clearly not identical. Additionally, most of the neuropsychological tools which are used for the evaluation of psychomotor and mental speed, also assess other neurocognitive functions, and this is at least partially a consequence of a methodology effect, because in order to measure ‘speed’, you need to initiate a ‘procedure’ whose ‘speed’ is going to be measured.

It has been reported that the reaction time in bipolar depressive patients, is prolonged and associated with burden of illness and especially past history of depressions but not with current medication [26–30].

The deficit could be present already during the early stages of BD [31]. Individual studies, suggested that the magnitude of mental speed impairment in patients with BD is reported to correspond to an effect size of 0.82–1.08 [21, 32, 33]. When all phases of the illness are taken into consideration, the effect size 0.50–0.55 (which is similar to that observed concerning the IQ) [24, 25].

## Attention

Attention is a concept that includes a number of processes which work together, influence one another or prerequisite one another. These processes are: working memory (which refers to the ability to keep a limited number of mental objects in awareness for a limited duration of time), vigilance (which is the capacity to identify a specific target among many other stimuli), freedom from distraction or interference and the ability to split or to rapidly shift attention. Concentration is a term which refers to the ability to sustain attention over prolonged periods of time. There are many tests, with each of them assessing one of the previously mentioned processes. For example, the Continuous Performance Test assesses vigilance while ‘span tasks’ assess working memory. However, all these tests except from the specific aspect of attention they assess, are also influenced from the other processes which are related to attention as well. Working memory is often classified as belonging to the executive functions and it is often considered in relation to them.

It has been also reported that the impairment is present already during the early stages of the disorder [31]. A number of meta-analyses suggest that the effect size calculated after a meta-analysis varies between 0.41 and 0.90 depending on the sub-domain assessed [24, 34–38].

## Learning and memory

Learning refers to the ability to acquire and store new information. Memory is the mental process that allows individuals to retrieve the new information at a later time. Learning and memory involve a number of processes including attention and concentration, encoding and allocation of effort. These processes are distinct from one another but interrelated and interdependent. Moreover, there are different strategies and processes involved, depending on whether a short- or a long-term effect is desirable and also depending on the quality and nature of the information and the frame it is presented in. Due to the fact that much of research on memory is focused on ‘depression’ and does not distinguish between unipolar and bipolar depression, the results and the conclusions from these studies should be received with reservation, because it is uncertain whether they apply specifically to BD, and to which extend.

Deficits in all facets of memory have been reported in patients with BD [27, 28, 39–43]. It has been suggested that most memory impairments are due to the presence of confounding variables except maybe for verbal recall [44]. The same is true concerning most aspects of learning [39, 45–49].

In meta-analytic studies, when all phases of the illness are taken into consideration, the magnitude of the effect size is 0.60 for working memory, 0.43 for immediate verbal memory and 0.34 for delayed, 0.26 for immediate visual memory and 0.51 for delayed [24, 25, 34, 50, 51].

## Verbal skills

The evaluation of verbal skills, includes mostly the evaluation of verbal fluency. Although the literature has reported that verbal skills are impaired during all phases of BD [27, 47, 52], patients with psychotic BD manifest a worse performance with a higher effect size (0.68–1.73) [32, 53, 54]. In general, when all phases of the illness are taken into consideration an effect size equal to 0.63 emerges [25]. Both letter fluency and semantic fluency are impaired during the acute manic/mixed state [34].

## Visuospatial skills

Patients with BD and their unaffected relatives show impairment in the visuospatial/constructional abilities [55–57] and in visual learning and memory [57, 58].

## Executive function

The executive system is considered to be involved in the planning, the decision-making, the error correction and the troubleshooting, in situations where responses are not well-rehearsed or contain novel sequences of actions, are dangerous or constitute technically difficult situations or situations requiring the overcoming of a strong habitual response or resisting temptation. In other words, ‘Controlling of mental and neurocognitive processes’ seems to be the key phrase describing the role of executive functions. In patients with BD, reasoning should be considered separately from the rest executive functions due to the fact that it seems to rely heavily on verbal and linguistic skills [24].

A severe impairment in executive functions except reasoning during all phases of BD is generally reported [28, 59–61] and it might be particularly severe concerning interference and inhibitory control [62–65].

Meta-analyses suggested that when taking all phases of the illness together, the effect size of this impairment is equal to 0.34–0.99 depending on the aspect of the cognitive function under investigation and the phase of the illness [24, 25, 34, 37, 38, 66, 67].

## Social cognition and theory of mind (ToM)

The term ‘Social cognition’ constitutes a psychological domain with several dimensions. It refers not only to the ability of the person to assume that other people have minds similar to his/her own and to interpret, but also to understand and predict the emotions, desires, intentions, behaviors and speech of others (including nonverbal elements). Social cognition shapes communication and interaction with others and in this way enabling adaptive social adaptation. It involves a complex set of processes including the representation of internal somatic states, knowledge about the self, perception of others, and interpersonal motivations.

The broad Theory of mind (ToM), includes three main processes (a narrow definition of ToM, emotion processing, and affective decision-making). The narrow definition of ToM (mentalizing or mindreading), refers to the ability to attribute mental states (e.g. beliefs, desires, and intents) to oneself and to others. Emotion processing is the ability to identify and discriminate basic emotions. Affective decision-making is crucial for an appropriate social behavior, and concerns weighing up choices in association with reward and punishment.

The tests which are used to evaluate these domains are both verbal (scenarios) and nonverbal (pictures). They demand the subject to identify and comprehend the situation, the roles and the interactions and to make appropriate planning. So far, empirical data have confirmed the universality of facial emotions. This means that the specific ability to process and identify facial emotions is a substantial feature of human communication and social interaction, which is independent of culture.

Generally, the literature supports the presence of a robust deficit in ToM in BD patients [68–75]. This deficit seems restricted to the acute phases of the illness even when memory was controlled for [39] and thus there is no impairment during remission [76].

There are inconclusive data concerning the recognition of emotions in BD patients, and there is little or no difference between patients with BD and controls on the emotional decision-making component [10, 77, 78].

An effect size concerning ToM equal to 0.75–0.86 and concerning emotion processing equal to 0.35 was reported [79].

## Clinical correlations

Medication constitutes an important confounding variable when comparing the different phases of BD. Some acutely ill patients might be medication-free during testing, however, this is not the case with patients in remission. As a result, medication status not only constitutes a confounding variable which is difficult to control for, but also might introduce a bias towards the detection of a deficit, especially in patients in remission. On the other hand, however, patients with severe mania or severe depression cannot be tested and are rarely off medication. Medication could be a possible reason why patients with BD have poor performance on certain neurocognitive tasks. This is in accord with the traditional concept that BD is considered to belong to the ‘functional psychoses’. According to this approach, the attentional impairment is considered to be the core neurocognitive deficit and the cause of all other deficits in neurocognition.

Patients under lithium often report that lithium inhibits their productivity and creativity [80]. Additionally, it seems that lithium has a negative impact on neurocognition especially on memory and psychomotor functioning [81, 82]. Lithium also causes a deficit in the long-term recall (retrieval) without having an effect on attention or on encoding. This impairment might especially concern verbal memory [32, 83].

The data on the possible deleterious effect of antipsychotics and antiepileptics on neurocognition are rare and conflicting [84–86]. Even after controlling for clinical features, current antipsychotic treatment is related to worse performance across all executive function tests as well as in verbal learning and recognition memory and in semantic fluency in BD patients [50, 87, 88].

Overall, it has been shown that medications have a limited adverse effect on neurocognitive function [89] if any at all [90–92].

It has been shown that both the presence of psychotic symptoms [93–97] as well as the history of psychotic features are also strongly related to a worse neurocognitive performance [94, 98, 99].

It is interesting that the neurocognitive impairment is present during all phases of BD. A meta-analysis, calculated the respected effect sizes for specific neurocognitive domains separately for each phase. During the acute manic/mixed states, these effect sizes showed a clear impairment in attention (0.79–0.90), verbal learning (1.43) and delayed free verbal recall (1.05), letter fluency (0.51) and semantic fluency (0.59), general executive function (0.72) and speeded set-shifting (0.64). During acute bipolar depression, there were impairments in attention (0.80), verbal memory (1.20), phonemic fluency (0.93) and executive function in speeded set-shifting (0.64). During the euthymic phase, these effect sizes showed a clear impairment in auditory (0.41) and sustained visual vigilance (0.69) and speeded visual scanning (0.65), working memory (0.65), verbal learning (0.81) and long-delay verbal free recall (0.78), executive functions concerning problem-solving tasks (0.54), verbal interference (0.75) and set-switching tasks (0.73), immediate nonverbal memory (0.73), delayed nonverbal recall (0.80), visuospatial function (0.55), phonemic (0.51), and semantic (0.75) verbal fluency and finally in psychomotor speed (0.66) [34]. Overall, these results suggested that patients in a manic or depressed state had significantly greater effect size impairment in verbal learning than patients in an euthymic state.

Important is to note that the overall evidence suggests that the observed neurocognitive deficit in BD patients is not secondary and does not constitute a by-product of mood symptomatology or of exposure to medication. This is in spite of the observed strong relationship between mood symptoms and neurocognitive impairment. The most probable explanation is that neurocognitive impairment reflects a deeper neurobiological dysfunction which probably includes the presence of premorbid developmental abnormalities [100].

Although the prevailing opinion is that BD-II patients perform better than BD-I but worse than healthy controls the literature has shown that in quantitative terms, BD-II does not differ much from BD-I [101–104].

## Long-term development of the neurocognitive deficit

According to Kraepelin, BD was originally considered to be an episodic illness with a rather benign course and outcome, but this is no longer the case.

**Fig. 1:**
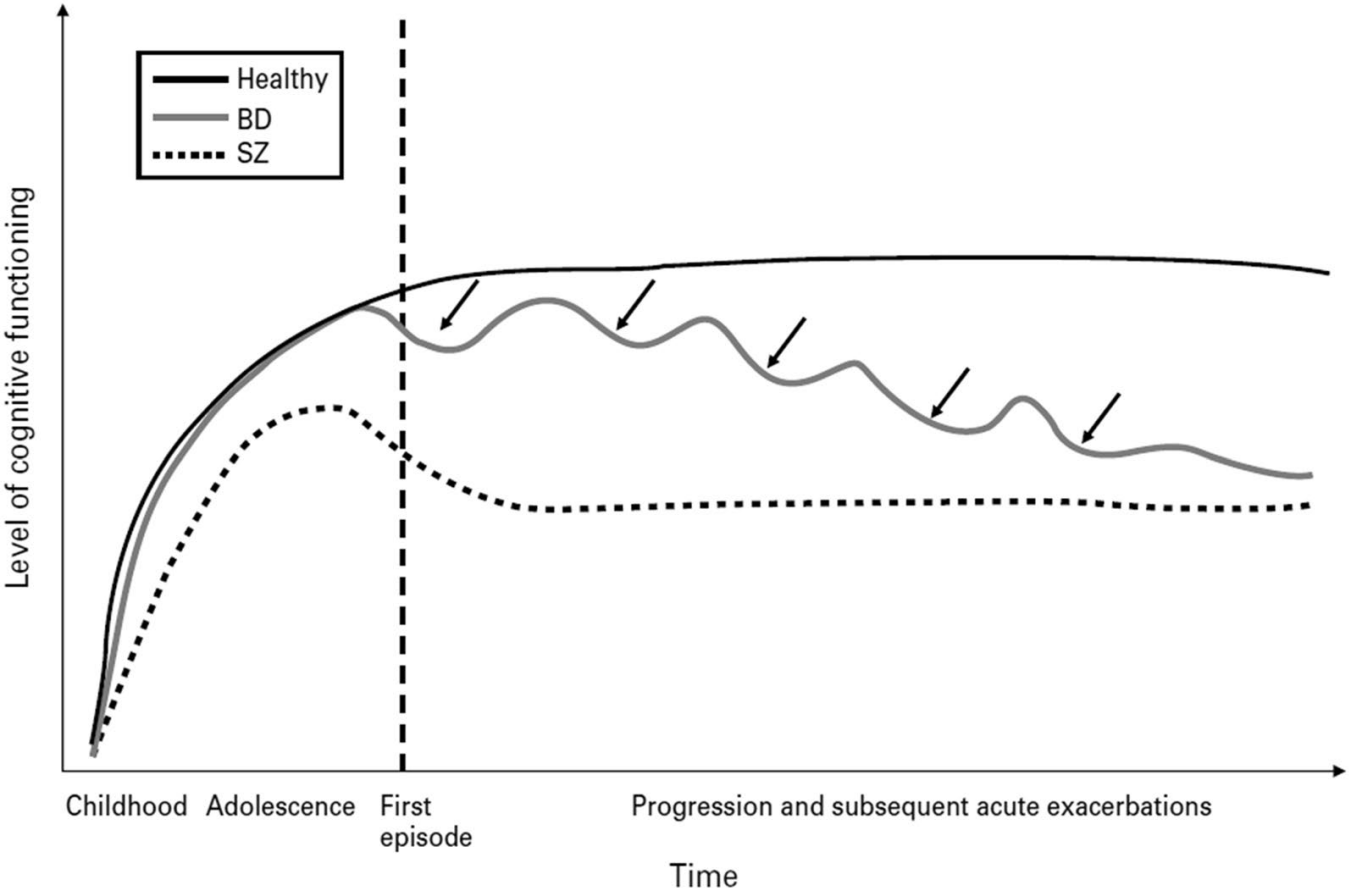
Long-term evolution of the neurocognitive deficit in BD patients, in comparison to patients with schizophrenia and normal subjects. Overall, in contrast to schizophrenia patients, BD patients exhibit a relatively intact cognitive functioning throughout childhood and adolescence, and the neurocognitive deterioration is observed only after the overt symptom onset (Reproduced after permission from Lewandowski et al. [105])

The overall longitudinal course suggests that neurodevelopmental factors play a minor role in the emergence of neuropsychological dysfunction in BD [100, 105] but opposite findings do exist [106]. Recent findings suggest that its course is often characterized by chronicity and residual symptoms with lack of remission between episodes, and possibly there is a progressive neurocognitive deficit. In this frame, an important role is attributed to the neurocognitive impairment in the specific clinical course of BD [107, 108]. As previously mentioned, there is a deleterious effect of psychotic symptoms [105] but otherwise the data in favor of a neurodegeneration are not consistent, and this is why the progression is characterized as ‘possible’ [109, 110]. It seems that eventually BD patients come to manifest a neurocognitive deficit similar in qualitative as well as in quantitative terms to patients with schizophrenia (Fig. 1).

The neurocognitive deficit is largely responsible for functional disability [111] and it is important to note that objective and not perceived deficit is what correlates with disability [112].

A graphical representation of the long-term development of the neurocognitive deficit in BD patients in comparison to schizophrenia and normal controls is shown in Fig. 1.

## Awareness of the neurocognitive deficit

Although many patients with BD complain frequently about neurocognitive problems in attention, concentration and memory, there are limited data on the relationship between subjective cognitive complaints with objective neuropsychological deficits.

The patients’ subjective cognitive complaints do not seem to correlate or predict objective neuropsychological deficits [113–115] or disability [112]. There are some possibility mood symptoms to mediate lack of awareness but currently, this is a matter of debate [116].

## Treatment of the neurocognitive deficit

It is important to assess the patient in a precise way and locate the areas of neurocognitive impairment so that monitoring of response is possible. Even patients in full remission should be assessed in detail [117].

There some but limited hard data concerning the efficacy of psychopharmacological agents in the treatment of neurocognitive deficit [118, 119]. The literature suggests that galantamine may have specific benefits for episodic memory, but not processing speed, in patients with cognitive impairment as part of BD [120]. Pramipexole may improve neurocognition in euthymic patients only [121], while the data are negative for N-acetyl cysteine (NAC) [122]. Adjunctive intranasal insulin (40 IU q.i.d.; N = 34) had a beneficial effect on executive function but not on the other neurocognitive measures in euthymic patients [123]. Also adjunctive mifepristone, which is a synthetic steroid, at 600 mg/day improved spatial working memory in BD depressed patients, and this was evident also 7 weeks after the cessation of treatment [124]. A randomized, open-label, pilot study, on euthymic BD-I patients supported the use of adjunctive lurasidone adjunctive in improving cognition [125]. A more recent study reported that six intravenous infusions of ketamine (0.5 mg/kg) over a 12-day period improved the speed of processing and verbal learning but it seemed that the improvement in depression mediated these results, with better baseline neurocognitive function being predictive of a better outcome [126].

There are also some limited data in support of the usefulness of non-pharmacological biological treatments. ECT was reported to produce an improvement in neurocognitive function similar to that of algorithm-based pharmacological treatment [127]. A recent study reported that ten consecutive days of high-frequency active rTMS improved aspects of neurocognition without an effect on mood symptoms [128].

Concerning psychotherapies, the most often used methods are Cognitive Remediation and Functional Remediation. Overall, the data are positive in improving functioning, which is the critical endpoint, but mostly negative concerning cognitive outcomes when using cognitive remediation techniques as add on to TAU in BD patients [129–132], although a more recent post hoc analysis was promising [133].

A recent systematic review identified eleven articles reporting data on Cognitive and Functional Remediation from seven original studies encompassing 471 participants. These authors concluded that methodological quality was problematic and there was a moderate risk of bias. While the majority of studies reported a beneficial effect, the findings were isolated and not replicated across studies, making any conclusions problematic [134].

A recent study reported that Functional Remediation had no effect in serum brain derived neurotrophic factor (BDNF) levels in euthymic adult BD patients despite the improvement in psychosocial functioning [135].

## Discussion

The current paper briefly summarized the view of the author concerning the literature on the neurocognitive deficit and its treatment in BD patients [9, 10]. The area is understudied, but the data so far suggest that neurocognition in BD is an important issue, the deficit is qualitatively similar but quantitatively milder in comparison to schizophrenia, it is present already since the first episode, is weakly related to mood symptoms and somewhat stronger to psychosis, it probably determines much of the disability associated with BD and treatment is problematic. This deficit is also present during periods of euthymia and the possible adverse effect of psychotropic medication is rather small if any at all and is confounded by the specific clinical symptoms, for which medication is used for their treatment. This is especially true concerning antipsychotics and psychotic symptoms.

The core deficit, which constitutes a direct consequence of the disease itself, seems to be relatively (but not completely) independent not only from the other components of the disease but also from mood symptoms. This core deficit is either increased or on the contrary it is attenuated by many factors like the disease phase, specific personal characteristics of the patients (age, gender, education, etc.), current symptomatology and its treatment and the long-term course and the long-term exposure to medication, psychiatric and somatic comorbidity and alcohol and/or substance abuse.

However, the origin and the etiopathogenesis of the core neurocognitive impairment remain elusive. The presence of a neurodegenerative component as a consequence of repeated mood episodes and psychotic features and of a neurodevelopmental component has both data in favor and against and they are both the focus of debate. This probably differentiates BD from schizophrenia, in which the neurodevelopmental component is strong. Such a neurodevelopmental effect is evident in some but not all patients with BD.

Overall, the neurocognitive deficit concerns almost all domains with only a few exceptions and its magnitude is at the severe range during the acute episodes and at the medium range during euthymia. A summary of effect sizes by neurocognitive domain and illness phase is shown in Table 2 [9, 10].

**Table 2:** Effect sizes concerning the various neurocognitive domains during different phases of BD as well as in high risk relatives (endophenotypes)

Domain	All phases	Acute mania	Acute bipolar depression	Euthymia	Endophenotypes
Intelligence Quotient (IQ)					
Premorbid IQ	Normal				Normal
Current IQ	0.36–0.70	0.28–0.47		0.11–0.50	0.20
Psychomotor and mental speed	0.50–0.55			0.52–0.80	0.17–0.22
Attention	0.64	0.79–0.90	0.80	0.41–0.80	0.18–0.36
Memory					
Working memory	0.60			0.54–1.02	
Verbal memory					
Immediate	0.43			0.73–0.82	0.33–0.42
Delayed	0.34	1.05	1.20	0.71–0.85	0.27–0.33
Verbal learning	0.91	1.43		0.66–0.90	0.28
Nonverbal memory					
Immediate	0.26			0.73	
Delayed	0.51			0.62–0.80	0.13
Episodic memory				0.62	
Visuospatial function	0.65			0.22–0.57	
Language/verbal fluency	0.63	0.51–0.59	0.93	0.34–0.90	0.27
Executive function	0.34–0.79	0.64–0.72	0.54–0.75	0.52–0.88	0.24–0.51
Social cognition					
ToM	0.75–0.86				
Emotion recognition	0.35				
Emotion decision-making	Normal				

The range of values reflects heterogeneity in study samples but also heterogeneity because of the different neuropsychological tools used

The specific clinical course of the illness seems to play an important role. A course characterized by residual symptoms, chronicity and lack of remission between episodes is related to a progressive neurocognitive deficit. Of significant importance is the adverse effect specifically of psychotic symptoms, while manic episodes seem to affect neurocognitive function more than depressive episodes do. Age, age at onset, duration of the illness and number of episodes, not only reflect distinct but overlapping aspects of the overall disease burden but also are related to the neurocognitive impairment and its progression. However, the data are inconclusive concerning the magnitude and the true nature of this relationship.

## Conclusion

The neurocognitive deficit in BD is relatively (but not completely) independent not only from the other components of the disease but also from mood symptoms. Its treatment and the restoration of functioning are problematic. Not only the data are limited but it seems that treatment options are few and with a weak overall effect. Pharmacological treatments, ECT and rTMS present some hard data, while the literature is inconclusive concerning psychotherapeutic interventions.

## Data Availability

Not applicable.
